# Diagnosis of *Haemophilus influenzae* Pneumonia by Nanopore 16S Amplicon Sequencing of Sputum

**DOI:** 10.3201/eid2410.180234

**Published:** 2018-10

**Authors:** Jangsup Moon, Yoonhyuk Jang, Narae Kim, Wan Beom Park, Kyung-Il Park, Soon-Tae Lee, Keun-Hwa Jung, Manho Kim, Sang Kun Lee, Kon Chu

**Affiliations:** Seoul National University Hospital, Seoul, South Korea (J. Moon, Y. Jang, N. Kim, W.B. Park, S.-T. Lee, K.-H. Jung, M. Kim, S.K. Lee, K. Chu);; Seoul National University Hospital Healthcare System Gangnam Center, Seoul (K.-I. Park)

**Keywords:** 16S amplicon sequencing, culture negative pneumonia, H. influenzae pneumonia, bacteria, South Korea, pneumonia

## Abstract

We used deep sequencing of the 16S rRNA gene from sputum to identify *Haemophilus influenza* in a patient with community-acquired pneumonia. This method may be more effective than conventional diagnostic tests in pneumonia patients because of its speed and sensitivity.

Pathogen identification in patients with community-acquired pneumonia primarily relies on culture-based techniques (*1*,*2*). Sequencing-based approaches for pathogen identification are being applied to pneumonia patients (*3*). MinION (Oxford Nanopore Technologies, Oxford, UK), a nanopore sequencer, is gaining attention in metagenomics research because of its capability for long-read sequencing and real-time analysis, along with its small size (*4*,*5*). Recently, the first use of MinION for real-time metagenomic sequencing of bronchoalveolar lavage (BAL) specimens in pneumonia patients was reported (*6*). We report successfully detecting a respiratory pathogen by deep sequencing of 16S amplicons of sputum using MinION.

A 77-year-old man with end-stage renal disease and asthma was hospitalized in June 2017 because of hypoxic respiratory failure. Dyspnea developed 4 days before admission, and sputum production and rhinorrhea increased significantly. Crackles were present in both lungs, and tachypnea was noted. Chest computed tomography scan revealed multiple nodular lesions and branching opacities in both lungs (Figure, panel A). Leukocytosis was absent, but C-reactive protein and procalcitonin were elevated (46.41 mg/dL [reference 0–0.5 mg/dL] and 32.03 ng/mL [reference 0–0.5 ng/mL], respectively). Results of extensive diagnostic testing performed on sputum, including Gram staining, bacterial culture, acid-fast bacilli testing, and PCR for 16 respiratory viruses and tuberculosis/nontuberculous mycobacteria, were negative. After 2 weeks of empiric antimicrobial treatment with ceftazidime and ciprofloxacin, the patient recovered to baseline status.

**Figure:**
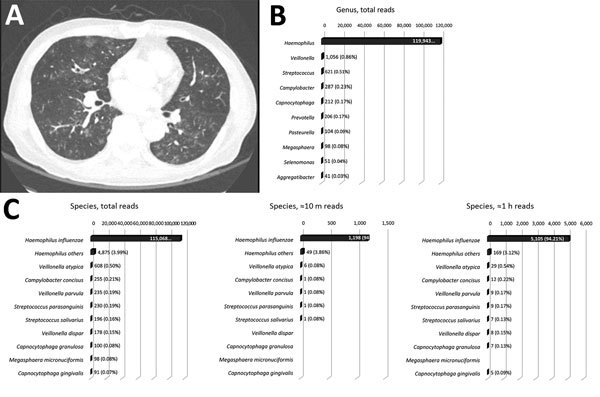
Chest computed tomography scan and sequencing of the 16S amplicon in a 77-year-old man with end-stage renal disease and asthma. A) Hypoxic respiratory failure with bilateral infiltrates are visible on chest computed tomography scan. B) Sequencing of the 16S amplicon performed on sputum using the MinION sequencer (Oxford Nanopore Technologies, Oxford, UK). Sequencing for 5 h generated 470,231 reads. A total of 122,272 reads were aligned with 1 of the bacterial 16S rRNA gene sequences, and most reads (119,943 [98.1%]) were aligned with genus *Haemophilus*. C) Of the 122,272 aligned reads, nearly all (115,068 [94.11%]) were aligned with the species *H. influenzae* (left). The number of reads aligned with *H. influenzae* was >100-fold larger than those aligned with other oral commensal bacteria. Similar results were obtained from the subgroup analyses of reads generated during the first hour (middle) and during the first 10 min (right).

We retrospectively performed 16S amplicon sequencing with MinION. We extracted genomic DNA (Genomic DNA Mini Kit, Invitrogen, Carlsbad, CA, USA) from sputum obtained by oropharyngeal suction after a single empiric administration of an antimicrobial drug (cefuroxime, 500 mg). We generated the sequencing libraries using a rapid 16S amplicon sequencing kit (SQK-RAS201). After 30 cycles of PCR using universal 16S primers (27F and 1492R) included in the kit, we attached sequencing adaptors. A total of 470,231 reads were generated during the 5-hour sequencing time. We analyzed the reads using the EPI2ME 16S BLAST workflow (https://blast.ncbi.nlm.nih.gov/Blast.cgi); 122,722 reads aligned with 1 of the bacterial 16S rRNA gene sequences with >80% accuracy. Of these reads, 119,943 (98.1%) were aligned with the genus *Haemophilus* and 115,068 (94.11%) were aligned with *Haemophilus influenzae* (Figure, panels B, C). We obtained similar results by analyzing the subgroups of reads generated during the first 10 minutes and during the first hour (Figure, panel C). Because the overwhelming majority of the reads were aligned with *H. influenzae* versus other oral commensal bacteria, we regarded *H. influenzae* as the pathogen. Repeated nanopore sequencing using different workflow and additional quantitative PCR confirmed the results (Technical Appendix).

We identified the pneumonia pathogen in this patient by deep sequencing of 16S amplicons from sputum using MinION. The reads aligned to *H. influenzae* were >100-fold more abundant than reads aligned with other commensal bacteria, reflecting the significant proliferation of *H. influenzae* in the patient’s respiratory tract. *H. influenzae* is an opportunistic pathogen of the respiratory tract that becomes pathogenic only when other risk factors are present (*7*). *H. influenzae* infection is most effectively treated with intravenous third-generation cephalosporins, whereas resistance to β-lactam antimicrobial drugs is prevalent (*8*).

We suggest deep sequencing of the 16S rRNA gene from sputum as a new method of detecting respiratory pathogens. Although expectorated sputum is the most readily available specimen, the specimen must transverse the upper airways, which are colonized with multiple bacteria; thus, criteria for acceptable sputum are widely used (*9*). Otherwise, quantitative cultures of BAL specimens are used; these specimens are less affected by upper airway commensals, but BAL is largely restricted to nosocomial or ventilator-associated pneumonia (*10*). Respiratory pathogens can be identified directly from sputum by comparing the relative ratio of reads aligned with each bacteria, without the prerequisite of microscopic examination or bronchoscopy.

Nanopore sequencing of 16S amplicons enables rapid pathogen identification in pneumonia patients. With the MinION sequencer, generated reads can be analyzed in real time, which makes this approach more promising (*4*,*6*). Tentative point-of-care diagnosis by nanopore 16S sequencing and confirmation of the result by standard culture methods would be a feasible approach. In the case we report, we performed sequencing for 5 hours; moreover, the subgroup analyses of reads generated for the first hour and for the first 10 minutes produced similar results, indicating that a relatively short sequencing time would be sufficient for pathogen identification. We estimate that the turnaround time for MinION 16S sequencing can be reduced to <8 hours.

The 16S amplicon sequencing–based diagnostic approach can be more sensitive than conventional tests and would be particularly useful for identifying unculturable bacteria or detecting bacteria in specimens collected after exposure to antimicrobial drugs. Therefore, this method might enable detection of pathogens that were not detected by conventional tests (*3*), as demonstrated by the case we report.

Nanopore 16S amplicon sequencing from sputum can be more effective than conventional diagnostic tests in pneumonia patients because of its speed and sensitivity. However, further studies with more cases are needed to establish reliable diagnostic criteria for respiratory pathogens based on the relative read abundance compared with commensal bacteria.

Technical Appendix Significant abundance of *Haemophilus influenzae* confirmed by quantitative PCR; predominance of *H. influenzae* confirmed by repeated nanopore sequencing.
