# Supplementary material for: Diagnosis of Haemophilus influenzae Pneumonia by Nanopore 16S Amplicon Sequencing of Sputum
Source: Emerg Infect Dis. 2018 Oct;24(10):1944–6. doi: 10.3201/eid2410.180234 (PMC6154133; doi:10.3201/eid2410.180234)
Supplement: Technical Appendix — Significant abundance of Haemophilus influenzae confirmed by quantitative PCR; predominance of H. influenzae confirmed by repeated nanopore sequencing. [file 18-0234-Techapp-s1.pdf]

Article DOI: https://doi.org/10.3201/eid2410.180234

Diagnosis of *Haemophilus influenzae* Pneumonia by Nanopore 16S Amplicon Sequencing of Sputum

Technical Appendix

Technical Appendix Table. Significant abundance of *Haemophilus influenzae* confirmed by quantitative PCR*

Target	Primer or probe name	Nucleotide sequence, 5′–3′)	Mean C_t	dC_t	2 dC_t
H. influenzae, hpd†			28.709	−6.584	95.936
	hpdF822	GGTTAAATATGCCGATGGTGTTG			
	hpdR952	TGCATCTTTACGCACGGTGTA			
	Pb896i	[FAM]TTGTGTACACTCCGT[BHQ1-dT]GGTAAAAGAACTTGCAC[SpC6]			
Streptoccocus salivarius, GtfP‡			35.293		
	GtfP-F	CACGCCATGCTGGAAGTG			
	GtfP-R	GCGATGAGCCAAGCTGAAG			
	GtfP-Probe	[FAM]TTAGCTGCTGCGTAGACTTCGTCT[BHQ1]			

*S. salivarius was selected from the oral commensals as a representative strain. H. influenzae was >95 times more abundant than S. salivarius in the sputum. dCt, delta Ct value.
†The primer and probe sequences were obtained from World Health Organization recommendations (*1*).
‡The primer and probe sequences were obtained from a previous report (*2*).

References

1. World Health Organization. Laboratory methods for the diagnosis of meningitis caused by *Neisseria meningitidis*, *Streptococcus pneumoniae*, and *Haemophilus influenzae*. 2nd ed. Geneva: The Organization; 2011.

2. Srinivasan V, Gertz RE Jr, Shewmaker PL, Patrick S, Chitnis AS, O'Connell H, et al. Using PCR-based detection and genotyping to trace *Streptococcus salivarius* meningitis outbreak strain to oral flora of radiology physician assistant. PLoS One. 2012;7:e32169. PubMed http://dx.doi.org/10.1371/journal.pone.0032169

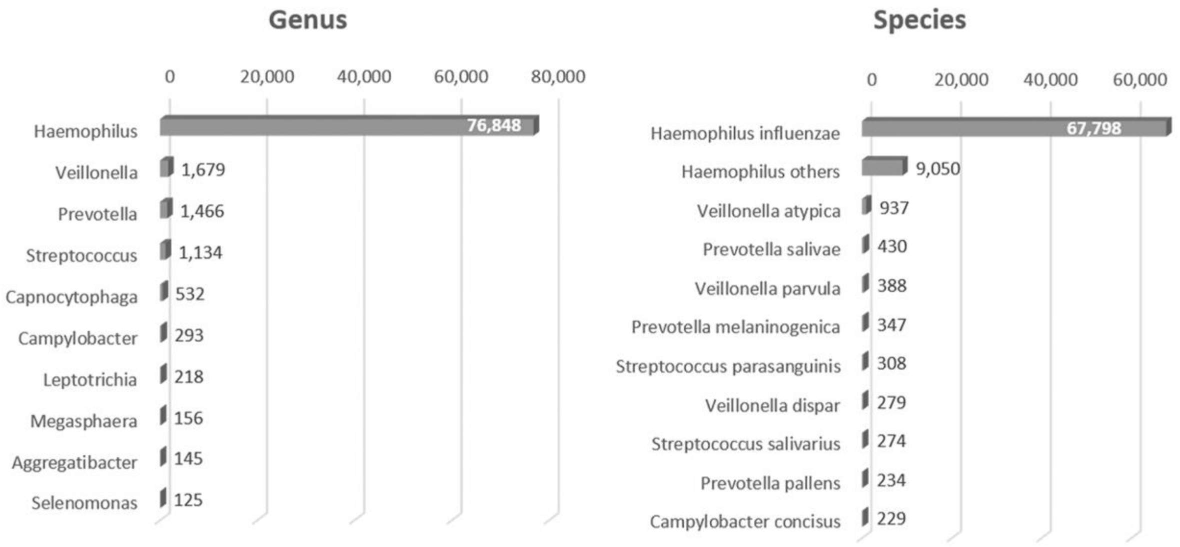

Technical Appendix Figure. Predominance of *Haemophilus influenzae* was confirmed by repeated nanopore sequencing. The 16S rRNA gene PCR was performed from the sputum DNA (16S rDNA Bacterial Identification PCR kit, TaKaRa, Kusatsu, Japan), following the manufacturer’s protocol. The sequencing library was generated from the PCR product using $1D^2$ sequencing kit (SQK-LSK308, Oxford Nanopore Technologies, Oxford, UK), which enables full-length 16S sequencing with higher accuracy. Sequencing was performed for 1 h and generated 166,127 reads. After the alignment of the reads to bacterial 16S rRNA gene sequences, *Haemophilus* and *H. influenzae* were the most prevalent genus and species, respectively. The number of reads aligned with *H. influenzae* was >70-fold larger than the number of reads aligned with other oral commensal bacteria
